# Interrelation of Sport Participation, Physical Activity, Social Capital and Mental Health in Disadvantaged Communities: A SEM-Analysis

**DOI:** 10.1371/journal.pone.0140196

**Published:** 2015-10-09

**Authors:** Mathieu Marlier, Delfien Van Dyck, Greet Cardon, Ilse De Bourdeaudhuij, Kathy Babiak, Annick Willem

**Affiliations:** 1 Department of Movement and Sports Sciences, Ghent University, Ghent, Belgium; 2 School of Kinesiology, University of Michigan, Ann Arbor, Michigan, United States of America; Örebro University, SWEDEN

## Abstract

**Background:**

The Health through Sport conceptual model links sport participation with physical, social and psychological outcomes and stresses the need for more understanding between these outcomes. The present study aims to uncover how sport participation, physical activity, social capital and mental health are interrelated by examining these outcomes in one model.

**Methods:**

A cross-sectional survey was conducted in nine disadvantaged communities in Antwerp (Belgium). Two hundred adults (aged 18–56) per community were randomly selected and visited at home to fill out a questionnaire on socio-demographics, sport participation, physical activity, social capital and mental health. A sample of 414 adults participated in the study.

**Results:**

Structural Equation Modeling analysis showed that sport participation (β = .095) and not total physical activity (β = .027) was associated with better mental health. No association was found between sport participation and community social capital (β = .009) or individual social capital (β = .045). Furthermore, only community social capital was linked with physical activity (β = .114), individual social capital was not (β = -.013). In contrast, only individual social capital was directly associated with mental health (β = .152), community social capital was not (β = .070).

**Conclusion:**

This study emphasizes the importance of sport participation and individual social capital to improve mental health in disadvantaged communities. It further gives a unique insight into the functionalities of how sport participation, physical activity, social capital and mental health are interrelated. Implications for policy are that cross-sector initiatives between the sport, social and health sector need to be supported as their outcomes are directly linked to one another.

## Introduction

Increasing rates of depression and low mental health are one of the most pressing problems of our society [1]. Sport participation, physical activity and social capital have been at the center of academic and policy interest for their positive effects on mental health [2–4]. Recently a conceptual model of Health through Sport has been conceived linking sport participation with social and psychological outcomes. The model includes three major elements: (a) sport participation, (b) determinants of sports participation, based on the socio-ecological model [5], (c) physical, social and psychological outcomes of sport participation [6]. Eime et al. articulate that more research should focus on investigating how sport, physical, social and psychological outcomes are associated [6]. The present study therefore aims to contribute to the existing literature by examining how sport participation, physical activity, social capital and mental health are interrelated. Incorporating these variables in one model enables insight into how they affect each other and which one is more important in increasing mental health. Having a better understanding of the complex interrelation of these variables should allow clarification of which activities could result in a multiplication of effects of physical, social and psychological outcomes. This study takes place in disadvantaged communities as mental health of residents in these communities is general worse [7], sport participation rates lower [8], physical activity levels inferior [9] and social capital standards lower [10] compared to those living in more prosperous communities. Moreover, action and research in these communities have been advocated to achieve greater health equity and to understand how this can be accomplished [11]. In following paragraphs a theoretical description is given of how these variables interrelate.

Sport participation and physical activity protect against and reduce symptoms of depression and anxiety, delay cognitive decline, increase self-esteem and feelings of energy, and contribute to the overall quality of life [2]. Mechanisms underpinning these association are partially allocated to physiological effects of aerobic exercise [12] and partially in psychological processes; (a) people being able to master difficult exercise tasks induce feelings of competence stimulating self-esteem (b) people with higher self-esteem and energy are believed to use more problem-focused coping strategies [13]. However, it is not clear yet how much physical activity is needed to improve mental health; findings about which type, duration, level or intensity of physical activity improves mental health, remain contradictory [14]. A large study in Europe reported different relationships across different nations in the European Union between physical activity and mental health [15]. In some studies, data suggested that there might exist a dose-response relationship, while in other studies this relationship could not be observed [15]. Of the different types of total physical activity (PA) (e.g., active transportation, leisure-time PA, household-related PA, work-related PA), leisure time PA has been found most related with higher levels of mental health [16]. In turn, of the different forms of leisure-time PA, sport participation has been consistently associated with better mental health in adults [4, 13, 17]. In this study sport participation is defined as physical activities that require a sufficient rate of exertion and that take place in an athletic context during leisure time [18]. It refers both to organized as well as non-organized and individual as team sport activities. The reason why sport participation is more closely related to higher levels of mental health has been assigned to intrinsic motivation to participate in sport as enjoyment and challenge which are key to an enhanced psychological well-being [19].

In recent years, research on the link between social capital and mental health has been stimulated by the growing recognition of social determinants of health [20]. These social determinants encompass among other poor social policies and bad access to education, health care, and leisure in the community [21], Interventions focusing on individual factors (knowledge, attitudes, skills) to improve health through behavioral change have resulted in limited effects, especially in disadvantaged populations [22]. In contrary, interventions focusing on social determinants of health have led to much better results [21]. Social capital has been acknowledged to reduce vulnerability to mental distress by impacting the social determinants, also in a disadvantaged context [23, 24]. Nevertheless, the concept of social capital is complex and much debated. Two main schools of thought are represented by Putnam and Bourdieu [25]. Putnam defines social capital as ‘features of social organizations, such as trust, norms, and networks’ (p. 67) [26]. Bourdieu’s definition is more focused on the resources that accrue to people as a result of participation in social networks [27]. As a consequence of these different views, it is essential for researchers to define how they conceptualize social capital. The literature discusses several different types of social capital which have different associations with mental health [3]. The most common distinction is made between cognitive and structural social capital [28]. The cognitive component refers to trust and reciprocity between individuals, whereas the structural component relates to the ties between friends, family and other social groups. Another debate in this field considers whether social capital is an individual or community-level construct or a combination of the two [29, 30]. Community social capital regards social capital as a collective attribute of the communities, which uniformly benefits all individuals living in that same community. Individual social capital in contrast, attributes the beneficial properties of social capital to the individuals and their social relationships [31]. The present study examines both cognitive community social capital and cognitive individual social capital. Only the cognitive type is investigated, as this type is most researched and consistently been related to positive mental health [3]. In contrast, the association between structural social capital and mental health remains ambiguous [3, 32]. In this study therefore community social capital refers to the trust and reciprocity one has of people in their immediate community [27, 33], and individual social capital refers to the trust and reciprocity one has of people in general [26, 34]. Some studies found that only individual social capital had better protective effects against mental illness [35, 36], while other studies detected that both community and individual social capital were related to better mental health [37, 38]. Concerning the relation between both types of social capital it is believed that higher levels of community social capital will boost individual social capital as people’s identity and behavior is partly shaped by their interactions with their social environment [39].

As previously mentioned, sport participation and mental health are closely related [2]. Sport participation has also been associated with social capital through participation in social and civic activities. Sports are considered a platform for people to meet, to enjoy being together and thus to create social networks [25, 30]. Furthermore, in many western countries, voluntary sport organizations make up the largest part of the voluntary sector [40]. According to most theorists, volunteering and active participation in civil society is a crucial element of social capital [25]. This has made the belief in the socially integrative effects of participation in sport and in voluntary organizations so strong, that it appears as self-evident [41]. Several authors warn however that the relation between sport and social capital is ambiguous. Coakley argues that this inherent belief in the purity and goodness of sport has been abused to sponsor sport events which contribute little to the common good in any representative manner [42]. Collins reasons that sport participation is exclusionary in itself as sport participation rates decline with lower socio-economic status [43]. Furthermore, studies have indicated that sport can also lead to inequalities and social exclusion as a result of the strong bonds that may exist within a sporting club or team that is homogeneous in its membership [44, 45]. The strong bonds may be beneficial to in-group members but negative for out-group members. It has therefore been argued that different types of sports and contexts where the sports take place are crucial for the social capital outcome [46, 47]. For instance, a study that focused on the relation of individual and organizational characteristics of sport clubs with social capital, found that members of team sports have stronger bonds with each other than in individual sports [41]. Another study in Japan showed that sport clubs open to people from all ages, from all levels providing various sports in the neighborhood scored higher on social capital compared to more traditional sport clubs, which were more focused on providing the technical practice of sport [46]. One context and type of sports activities which have been most explicitly linked with beneficial social and health outcomes are sport for development programs [47]. Many sport for development programs have recently been implemented in disadvantaged communities to reach United Nations Millennium Development Goals [48]. These programs use sport to exert a positive influence on public health, the socialization of children, youths and adults, the social inclusion of the disadvantaged, the economic development of regions and states, and on fostering intercultural exchange and conflict resolution (p. 311) [49].

The relationship between social capital and total physical activity still remains largely to be discovered [50]. Most studies that have investigated this relationship argue that both individual and community social capital are related to higher levels of physical activity [51, 52]. Their arguments are generally based on three mechanisms: (a) decline in crime rate which promotes perceptions of safety and consequently increases physical activity; (b) higher norms of health-related behavior which encourages residents to be more physically active; (c) higher collective efficacy among residents which improves access to resources for physical activity [52, 53]. This direction of the association between social capital and physical activity is reverse when compared with the previous argument regarding the relationship between sport participation (= predictor) and social capital (= outcome). However, total physical activity is much broader than sport participation only, so probably other types of physical activity such as active transportation, housekeeping, gardening and work-related physical activity interact differently with social capital, which could justify this reverse association.

**Fig 1:**
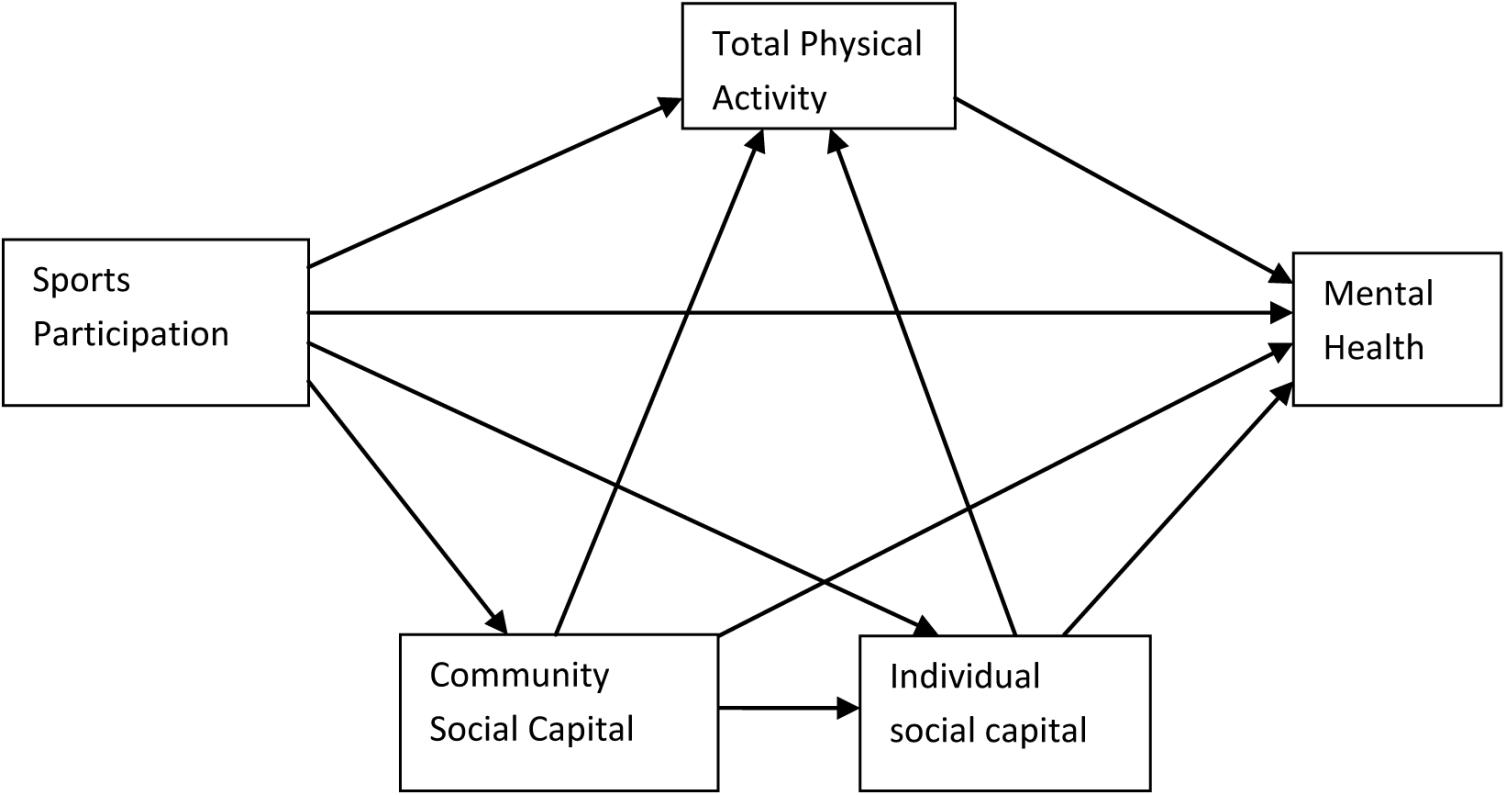
Hypothesized model of relationships between sport participation, total physical activity, community social capital, individual social capital, and mental health.

In conclusion, the Health through Sport conceptual model has indicated that sport is related to psychosocial outcomes and that this should be further investigated [6]. The present study aims to fill this gap by examining how sport participation, total physical activity, social capital and mental health are interrelated. This study differentiates from other studies by researching these associations in one model, enabling comparison of strength of associations between the different outcomes and measurement of indirect effects. Fig 1 represents the model that will be tested in this paper, showing hypothesized associations based on the results currently available in the literature. As a side note, the association between sport participation and total physical activity should not be regarded as a hypothesis, but rather as a fact. This subdivision has been made as a result of the different associations between sport participation and total physical activity with social capital and mental health, described in the previous section.

## Methods

### Participants

The study was conducted in Antwerp, Belgium (506,225 inhabitants, 204.26km², 2,478 inhabitants/km²), which is the city in Flanders (Belgium) where most disadvantaged communities are located [54]. Data were collected between January and March 2013. The study was approved by the Ethics Committee of the Ghent University Hospital and all respondents signed an informed consent. In the context of this study, disadvantaged communities were defined as ‘communities which suffer acute social problems such as increasing population densities, low socio-economic status, high rates of chronic disease, high levels of migration and multiculturalism and young people at risk of exclusion/disaffection from society’ (p. 264) [55]. Based on this definition four criteria were chosen for which data of the Public Service of Antwerp (2012) were available. For each of these categories the median was chosen as the cutoff point as this is the most common approach for dichotomizing continuous variables when no clear cutoff points are indicated by previous studies [56]. In total nine disadvantaged communities in Antwerp were selected based on four criteria: (1) average income (median declaration of net taxable income) lower than the city’s median of €19845; (2) unemployment rate (proportion of unemployed people looking for a job between 18 and 64 years) higher than city’s median of 8.9%; (3) ethnicity rate (percentage of parents born outside Belgium) higher than the city’s median of 30.0%; (4) population density (number of inhabitants per square kilometer), higher than 8005 inh/km². Two communities only met three out of four requirements but were still regarded as the best options when compared to other communities. The socio-economic characteristics of the selected communities of the Public Service of Antwerp are provided in S1 Table.

After the selection of the communities, potential respondents were selected. Prior power analysis indicated a total sample size of 400 adults living in the nine communities was needed. This implied that 45 respondents per community had to be included to have an equal sample distribution over the nine communities. Since recruiting respondents in disadvantaged communities presents itself as a complicated endeavor, a response rate of 25% was expected. The Public Service of Antwerp selected in each community a random sample of 200 addresses of adults (aged 18–56 years; 1800 adults in total) who had already resided more than two years in the community. Up to three attempts were made on different days (during the week and weekends) and different times (afternoon, evening) of the day to find these persons at home. Participating respondents were asked to complete a written informed consent. Researchers conducting the visits were able to fluently speak English and French next to Dutch, to assist if participants showed difficulties responding in any particular language. If language remained a barrier, the help of a family member or friend was asked to assist during the interview. Respondents were asked to answer survey questions on socio-demographics, physical activity, sport participation, social capital and mental health. As incentive to participate, nine city bikes (one per community) could be won. When people opened the door and did not want to participate, this was considered a rejection. When people did not open the door it was coded as ‘not at home’. People who were not home after three attempts, were not visited anymore. In most communities three rounds of home-visits were needed to recruit 45 participants.

### Measures

#### Socio-demographics

Participants were asked to give information about age, gender, education, ethnicity, tenancy, and civil status. Ethnicity was assessed by birth country of the respondents’ parents. These socio-demographic variables have been added to the model because evidence from both national and international literature suggests that sport participation [57], community social capital [58], individual social capital [58], total physical activity [59], and mental health [60] are differently distributed according to several of these socio-demographic characteristics. Moreover, the interaction effects of the socio-demographics have been added to the model, as socio-ecological models have emphasized the importance of interaction effects to explain health behaviors [5].

#### Sport Participation

Sport participation was assessed using the sport index of the Flemish Physical Activity Questionnaire (FPAQ) [61]. The criterion validity of this sport index, assessed against accelerometers was good with a *ρ* of 0.52 [62]. Respondents were asked to select up to three organized and non-organized sports they practiced. For each of these sports, data on frequency (from once a year to more than once a day) and duration (from some hours per year to more than 20 hours per week) was collected. Fluctuation of sport participation during different periods of the year was taken into account by questioning the number of months one practiced the sport throughout the year. A sport participation index was computed by summing hours per week spent in total for the different sports.

#### Total Physical activity

Self-reported total physical activity was collected using the short Dutch IPAQ (last seven days interview version). The interview version was chosen because adults tend to over report their physical activity levels with the self-administered version [63]. The short IPAQ has good reliability (intra-class range from 0.66 to 0.88). Criterion validity, assessed against accelerometers is fair-to-moderate with a median *ρ* = 0.29 [62]. Scoring was applied according to the guidelines of the short form IPAQ [64]. The metabolic equivalent (MET) values were derived for walking, moderate PA and vigorous PA and summed to create the total PA MET-minutes/week.

#### Social capital

**Fig 2:**
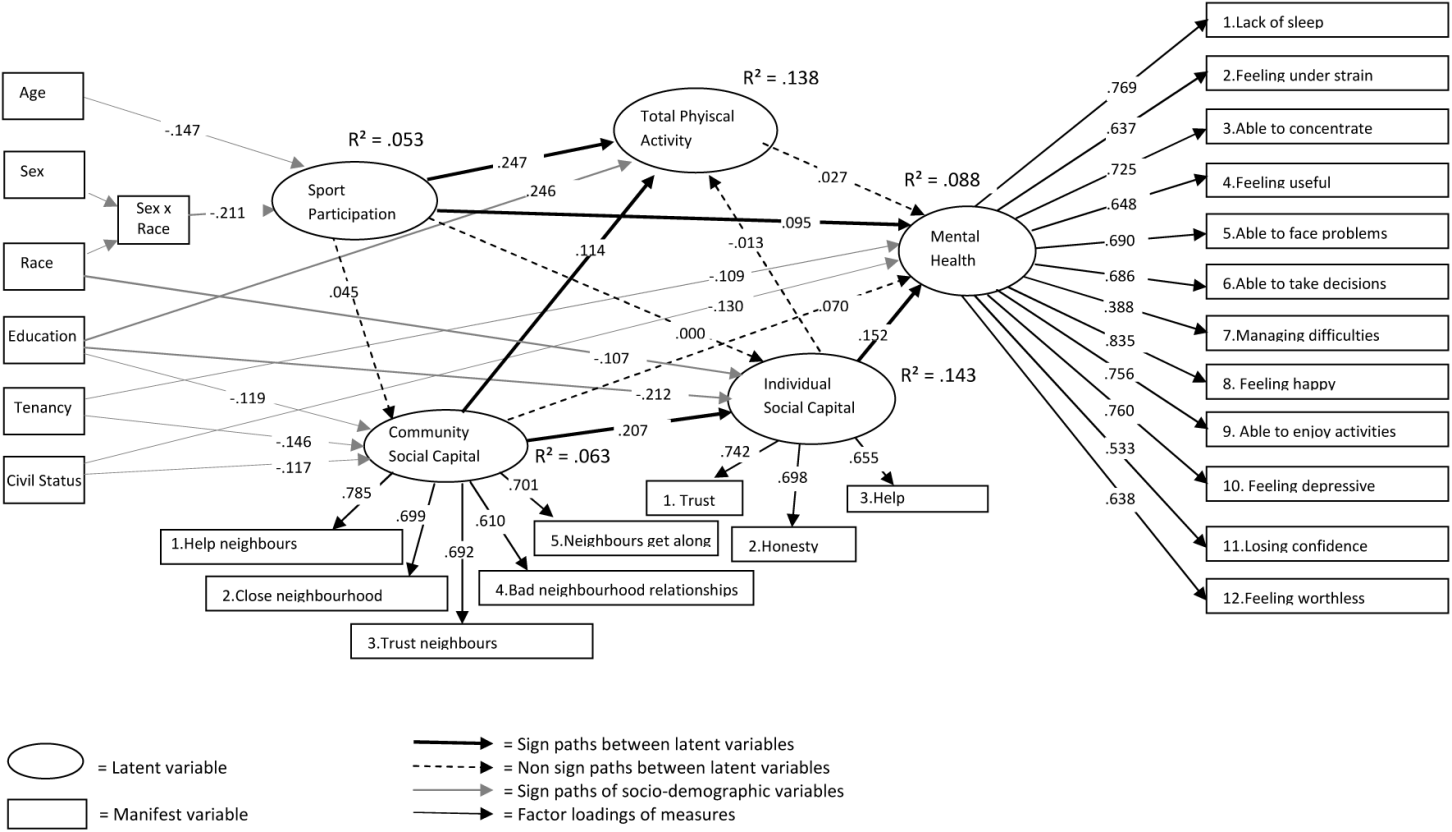
Structural equation analysis of sport participation, total physical activity, community social capital, individual social capital and mental health. Standardized parameter coefficients are shown.

To capture the multidimensionality of social capital both community and individual social capital were assessed. Community-level social capital was evaluated using a 5-item scale based on the theoretical work of Bourdieu [27] and further developed by Carpiano [33] (see Fig 2). An example item was: “People in this neighborhood are willing to help their neighbors?”. Five-point answer categories were applied (1 = strongly disagree, 2 = disagree, 3 = neither agree nor disagree, 4 = agree, 5 = strongly agree). The Cronbach’s alpha of the instrument in this study was 0.82.

Individual social capital was evaluated using a 3-item scale based on the ‘social capital community benchmark survey’ of Putnam [26]. Moreover, these items were core questions in the European Social Survey [34] (see Fig 2). An example item was: “Generally speaking, would you say that most people can be trusted, or that you can’t be too careful in dealing with people”. The questions had an 11-point answer scale ranging from 0 (e.g., you can never trust people) to 10 (e.g., you can always trust people). The Cronbach’s alpha of the instrument in this study was 0.73.

#### Mental Health

Mental health was measured using Goldberg’s General Health Questionnaire (GHQ-12) of [65]. The scale was a valid self-report instrument to assess a person’s wellbeing in the community and non-psychiatric clinical settings [66–68]. It consisted of 12 items (see Fig 2) with 4-point answer categories: ‘not at all’, ‘same as usual’, ‘rather more than usual’, or ‘much more than usual’. A sample item was: “Have you lately felt like you couldn’t overcome your difficulties?”. The bimodal GHQ-scoring method (1-1-0-0) was applied, as recommended by Goldberg [65]. The resulting total scores ranged from 0 to 12, with higher scores indicating higher perceived health and mental wellbeing. The Cronbach’s alpha of the instrument in this study was 0.83.

### Statistical analyses

MANOVA-models were conducted to determine socio-demographic differences in the latent variables of sport participation, physical activity, community social capital, individual social capital and mental health. Before estimation of the parameters, assumption of normality and equal factor loadings of latent variables were tested. First, concerning normality, sport participation and physical activity were positively skewed as is often the case with these variables. Therefore, skewness of physical activity and sport participation was improved with respectively a Log10 transformation and a Box-Cox transformation. Second, concerning equal factor loadings, the items of the scales of community social capital, individual social capital and mental health did not have equal contributions. As a result, factor loadings were used instead of summarized scales, to have more accurate estimates of community social capital, individual social capital and mental health. Factor loadings are depicted in Fig 2. Transformed variables were used to calculate F and p-values of MANOVA-analysis. To improve ease of interpretation however mean scores of raw data will be reported. Parameter estimates of the socio-demographics are shown in Table 1. This table does not show interaction effects; however, these are mentioned at the bottom of the table. These analyses were conducted with SPSS version 22.0 (IBM Corporation, Armonk, NY, USA).

**Table 1:** Mean of the raw scores of sport participation, total physical activity, average scores of community social capital, individual social capital and sum score of mental health for the different socio-demographic variables.

Groups	n (%)	Sport Participation (Hours/week)	*F-*value	Physical Activity (MET-minutes/week)	*F-*value	Community Social Capital (Range = 0–5) (0 = low 5 = high)	*F-*value	Individual social capital Range = 0-10(0 = low10 = high)	*F-*value	Mental Health Range = 0-12(0 = low12 = high)	*F-*value
**Age group**											
Young adults (18–37)	201 (48.6)	2.217 (3.636)		4799.677 (3512.898)		3.548 (.722)		5.877 (1.735)		9.924 (2.280)	
Older adults (38–56)	213 (51.4)	1.561 (3.018)	9.781**	4373.773 (3426.767)	.645	3.629 (.725)	1.201	5.766 (1.759)	.008	9.730 (2.505)	.872
**Gender**											
Men	189 (45.6)	2.293 (3.859)		4742.325 (3652.982)		3.628 (.684)		5.750 (1.714)		9.931 (2.303)	
Women	225 (54.4)	1.529 (2.800)	.4.546*	4443.187 (3312.155)	.042	3.558 (.756)	1.322	5.878 (1.775)	.021	9.973 (2.476)	.719
**Ethnicity**											
Native (parents born in Belgium)	222 (53.6)	1.782 (2.856)		4479.942 (3688.914)		3.658 (.673)		6.086 (1.688)		9.973 (2.528)	
Ethnic (parents born abroad)	192 (46.3)	1.990 (3.839)	2.172	4696.376 (3201.583)	.016	3511 (.774)	.823	5.510 (1.766)	5.627*	9.651 (2.230)	.1.010
**Education**											
College, university	194 (46.9)	1.718 (2.687)		3600.913 (2849.090)		3.702 (.665)		6.297 (1.516)		10.141 (2.123)	
Primary, secondary	220 (53.1)	2.019 (3.833)	2.680	5446.207 (3738.124)	20.347***	3.491 (.760)	4.427*	5.398 (1.830)	22.108***	9.544 (2.587)	2.973^+^
**Tenancy**											
Owner	267 (64.5)	1.758 (3.022)		4479.194 (3460.036)		3.685 (.690)		6.042 (1.583)		10.102 (2.109)	
No owner	147 (35.5)	2.099 (3.868)	.078	4765.388 (3495.612)	.011	3.415 (.754)	6.134*	5.412 (1.953)	3.923*	9.312 (2.790)	3.978*
**Civil Status**											
Married / stable partner	290 (70.0)	1.678 (2.927)		4502.681 (3477.808)		3.651 (.707)		5.888 (1.757)		10.039 (2.259)	
Single	124 (30.0)	2.313 (4.131)	.637	4759.637 (3462.652)	.526	3.449 (.746)	3.137^+^	5.661 (1.719)	.800	9.325 (2.635)	9.541**
**TOTAL**	**414 (100.0)**	1.868 (3.327)		4598.640 (3487.352)		3.585 (.724)		5.825 (1.741)		9.826 (2.393)	

P-values are calculated from the transformed variables of sports participation and physical activity and the factor scores of community social capital, individual social capital and mental health

^+^p<0.10

*p<0.05

**p<0.01

***p<0.001

Significant two-way interaction-effects are not mentioned in this table, these were

a) the interaction of gender and ethnicity with sport participation: F = 5.232 *

b) the interaction of gender and education with total physical activity: F = 4.056*

Structural equation modeling (SEM) was used to identify how sport participation, physical activity, community social capital, individual social capital and mental health (latent variables) were interrelated. SEM allows the simultaneous examination of a set of relationships between one or more independent variables and one or more dependent variables which makes it particularly useful to measure interrelationships of the latent variables set out in the hypothesized model of this study (Fig 1) [69]. Socio-demographic variables that were found to be related to the latent variables in the previous MANOVA-analyses were incorporated as covariates into the final model that tests the interrelations between the sport participation, community social capital, individual social capital, total physical activity and mental health variables. Not significant relations were discarded from the model. SEM-models were analyzed using MPLUS 7 (Muthen & Muthen). The bias-corrected bootstrap method (5,000 iterations) was used for measuring indirect effects and mediation as advocated by Preacher and Hayes [70].

To examine whether the hypothesized model fit the observed data, four indices were recommended as result of a lack of a standard format for reporting fit [71]: (a) the Root Mean Square Error of Approximation (RMSEA); a good fit is indicated when RMSEA is less than 0.05, (b) The Tacker- Lewis index (TLI) and comparative fit index (CFI); a good fit is indicated when TLI and CFI values are greater than 0.90, (c) the Standardized Root Mean Square Residual (SRMR); a good fit is indicated when SRMR is less than 0.05, (d) the normed Χ² chi square test, which is the chi-square fit index divided by degrees of freedom (this makes the test less dependent on sample size); a good fit is indicated when X²/df is less than 3 [69, 71]. If all indices demonstrate values close to or higher than the presented cutoff values, it is generally accepted that the model fits the observed data [72].

Finally multiple group analyses in SEM were executed to verify if relations in our structural model (presented in Fig 1), differed for male or female respondents, native or ethnic residents, high or low educated people and for other groupings of socio-demographic variables. The model was therefore fitted separately for the different groups of the socio-demographic variables. To assess whether differences between groups were significant, WALD-tests were completed [69].

## Results

From the 1800 randomly selected residents, 656 participants were found at home (36%). In total 242 declined to participate, resulting in a total of 414 valid questionnaires and a response rate of 63.1% (414 participants/656 participants found at home). The socio-demographic characteristics of the sample are presented in Table 1, a more detailed version of socio-demographic characteristics of the respondents per community can be retrieved in S2 Table. Although communities were selected on several criteria appropriate to disadvantaged communities, significant differences for the respondents of the communities were noted for ethnicity rate, education and tenancy. Meaning that some communities were more disadvantaged than others. In general, results showed that younger men participated more in sport; people with lower education had higher levels of physical activity; owners of a house and adults with higher education demonstrated higher levels of community and individual social capital; and married people and adults owning a house indicated having better mental health.

Subsequently, socio-demographic variables which were related to one of the latent variables were added as covariates in the SEM-analyses (i.e., for sport participation, age and gender were added; for physical activity, education was added; for community social capital, education, tenancy and civil status were added, for individual social capital ethnicity, education and tenancy were added; for mental health, civil status and tenancy were added).

Parameter estimates were calculated by a series of multiple regression analyses based on the hypothesized model (see Fig 1). The final model had a good fit with RMSEA = .000; CFI = 1.000; TLI = 1.000; SRMR = .021; x²/df = .702. The model showed that five out of ten of the initial hypotheses were confirmed. Fig 2 illustrates the model and its path estimates. Table 2 shows both the direct and indirect associations between sport participation, individual social capital, community social capital, total physical activity, and mental health. The interrelationship of the latent variables are presented below.

**Table 2:** Path coefficients for the direct and indirect associations for sport participation, community social capital, individual social capital, total physical activity and mental health.

Latent variables	Community Social Capital	Individual social capital	Physical Activity	Mental Health
**Sport Participation**				
Direct	.045	.000	.247***	.095*
Indirect	-	.009	.005	.011
Total	.045	.009	.252***	.107*
**Community Social Capital**				
Direct	-	.207***	.114*	.070
Indirect	-	-	-.003	.035*
Total	-	.207***	.111*	.105*
**Individual social capital**				
Direct	-	-	-.013	0.152**
Indirect	-	-	-	0.000
Total	-	-	-.013	0.152**
**Physical Activity**				
Direct	-	-	-	.027
Indirect	-	-	-	-
Total	-	-	-	.027

*p<0.05

**p<0.01

***p<0.001

### Sport participation

Sport participation had a direct association with total physical activity (β = .247; p < .001) and mental health (β = .095, p < .05). No direct associations were found between sport participation and community social capital (β = .045, p>.05) or individual social capital (β = .009, p>.05).

### Community social capital

Community social capital had a direct association with individual social capital (β = 0.247, p < .001) and physical activity (β = .114, p < .05) but not with mental health (β = .070, p>.05). However, a significant indirect association (β = .032, p < .05) of community social capital with mental health was discovered through individual social capital. Thus, individual social capital partially mediates the relation between community social capital and mental health. This made the total association of community social capital on mental health significant (β = .103, p < .05).

### Individual social capital

Individual social capital had a direct association with mental health (β = .152, p < .01), but no direct association was found with total physical activity (β = -.013, p>.05).

### Total physical activity

Total physical activity had no direct association with mental health (β = .027, p>.05).

Explained variances for the latent variables are depicted in Fig 2. Explanatory variables accounted for 5.3% of the variance in explaining sport participation, 13.8% of the variance in explaining physical activity, 6.3% in explaining community social capital, 14.3% in explaining individual social capital and 8.8% in explaining mental health.

Finally, findings of the multiple group analyses verified that in most cases the model did not differ between the different groupings of socio-demographic variables. This means that independent of being a man, or woman, high or low educated, married or single, the relations as presented in the model are valid. Two exceptions were noted. The first was a difference between native and ethnic respondents. Findings showed that higher levels of community social capital led to better mental health for native residents, whereas for ethnic residents this was not the case. The second was a difference between young and older adults. Results indicated that higher levels of sport participation led to better individual social capital for older residents, whereas for younger residents this was not the case. In S1 Appendix, details can be found for all multiple group analyses.

## Discussion

To our knowledge this is the first study to empirically examine the interrelatedness of sport participation, physical activity, social capital and mental health in one model. Six of the ten hypothesized relations (depicted in Fig 1) were confirmed. This study took place in disadvantaged communities where mental health condition of residents are known to be worse compared to those living in more prosperous communities [7].

One of the main findings was that sport participation and not total physical activity was associated with better mental wellbeing. This explains the ambiguous relationship of physical activity with mental health found in other studies [15]. Previous studies also concluded that sport participation and no other types of physical activity (e.g., active transportation, household- or work-related PA) is associated with better mental health [4, 13]. A plausible explanation for this result is that sport participation usually represents a chosen leisure-time activity aiming for recreation and enjoyment, which helps to improve mood and self-perception which are key to an enhanced psychological well-being [73]. This in contrast with other types of total physical activity as housekeeping, gardening and activity at work which rather imply compulsion [4]. For clarity purposes, we repeat that the used short version of the IPAQ used in this study did not allow us to differentiate between different types of physical activity. As such, we can only derive from our data that total physical activity does not relate with mental health. It is further important to note that other kinds of leisure-time physical activity (besides sport participation) (e.g., recreational walking) have also been associated with higher levels of mental health [74].

Surprisingly, results showed that sport participation did not have an association with any type of social capital, which is counter to the main claims in research that it operates as a platform for people to meet and create social networks [30]. According to Coakley this was to be expected as he has repeatedly stressed that social benefits of sport participation do not just happen, they need to be leveraged [42]. This finding might be a result of the tendency of respondents participating in sport in an isolated environment (e.g. running or working out alone), which disabled them from leveraging their social capital through sport [30]. In a post analysis 32.0% of our respondents, who indicated participating in sport, expressed that they did this in an isolated environment. In a subsequent ANOVA-analysis significant higher levels of both individual and community social capital were noted for people performing sport with friends and colleagues (when adding all other covariates however only a marginal significant difference remained). Other studies also concluded that sport in itself will not lead to better individual and community social capital, it is the social and organizational context that will determine if social capital is leveraged through sport participation [46, 47]. An interesting difference was found between younger (18–37 years old) and older residents (38–56 years old): for older residents higher levels of sport participation led to better individual social capital, whereas for younger residents this was not the case. A review of understanding participation in sport indicated that older adults mainly engaged in sport for reasons of social support, while younger people were more concerned about weight management [75]. It might be that older adults valued the social connection that sport provided more than younger adults did. In contrast, another study found that younger adults who participated in sports showed stronger social bonds compared to older adults [41]. In that study the sport context only included organized sports which might explain the different findings. Future studies investigating the relation between sport participation and social capital should therefore distinguish between socio-demographic characteristics (e.g. age), the social context (e.g. participating in sport activities alone or with other people) and the organizational context (e.g. organized, non-organized). Moreover, it would be especially interesting to see, if communities where sport for development programs aim explicitly at improving social capital of participants, show higher levels of individual and community social capital.

Respondents indicating higher community social capital had higher levels of total physical activity, concordant with results of another study [50]. No such relation however was found for adults reporting higher levels of individual social capital, which is partially counter to the findings of other studies [52, 53]. Explanations for these differences might be the consequence of different measures (i.e., one study measured physical inactivity rather than total physical activity [52], another used structural social capital rather than cognitive social capital [53]). Furthermore, the mechanisms offered by these studies relate to the effects social capital has on creating a stimulating environment to engage in total physical activity (i.e., better safety perception in the community, better health norms in the community, better collective efficacy in the community). These mechanisms are more related to the community and add credence to our findings that community social capital rather than individual social capital is important in increasing physical activity levels. Moreover, many physical activities take place in the immediate neighborhood, which adds importance to this argument [76]. However, in general little knowledge exists concerning the relationship between types of social capital and types of physical activity [50]. Future studies should therefore investigate how different types of social capital relate to different types of physical activity. Second, general studies have posited that social capital stimulates total physical activity [52]. However, several arguments can be found for the inverse relationship (i.e., that total physical activity fosters community social capital). For instance, people walking their dog, jogging in streets, running errands by bike or on foot are more likely to make contact with neighbors, which results in more connections and by doing so these people consequently foster higher social capital in their neighborhood. Studies incorporating a longitudinal design are needed to clarify the relationship between physical activity and social capital.

Consistent with previous studies, adults with higher levels of social capital reported better mental health [23, 24]. Individual social capital had a direct effect on mental health, whereas the effect of community social capital on mental health was partially mediated through its positive effect on individual social capital. This reaffirms results of other studies which concluded that individual social capital rather than community social capital is related with mental health[35, 36]. The core finding however was that individual social capital predicted mental health better than all other variables in the model. This study shows that even more substantial than being married or owning a house, the trust and reciprocity one has of people in general is most essential for better mental health. As this is the first study being able to compare the strength of relations with these variables, other studies will need to confirm or contradict this finding. Another interesting result was that higher levels of community social capital led to better mental health for native residents, whereas for ethnic residents this was not the case. To the best of our knowledge, no previous research has investigated this association. One explanation might be that for native people higher community social capital is more important to feel safe and to be able to go outdoors and interact with the neighbors, whereas for ethnic people a higher trust and feeling of reciprocity of the neighbors does not make them interact with the neighbors and does little to enhance their mental health. Future studies are needed to explain this relationship.

Apart from the significant indirect association of community social capital to mental health, no other significant indirect associations were noted. These indirect associations were mainly absent because only half of the direct associations between the different variables were significant. The explained variance of sport participation, total physical activity, social capital and mental health show that they played a significant role in explaining the variance of each other. However, it must be noted that for most variables about ninety percent of the variance remains to be explained. It would therefore be interesting to see in future studies how certain psycho-social factors and environmental factors would interact in the model. An interesting psycho-social factor to incorporate in the model would be social support from friends and family as this factor is known to be related to higher levels of sport participation, total physical activity, social capital and mental health [24, 77]. Interesting environmental variable to consider would be community crime rate and perception of safety as these interact both with total physical activity, community social capital and mental health [33, 52].

Finally, findings of the multiple group analyses added credibility to the tested model as in most cases the model did not differ between the different groupings of socio-demographic variables.

### Strengths and limitations

This study has three main strengths. The first is the incorporation of sport participation, total physical activity, social capital and mental health in one SEM-analysis, which enables us to explain the relationship and relative importance of each factor and to examine the direct and indirect relations among the variables. A second strength is the use of validated and reliable questionnaires to assess the latent variables. Finally, the study was conducted in disadvantaged communities. These communities are often understudied due to high time investment, low response rates and biased samples. The methodology of visiting respondents at home moderated these limitations.

Some limitations should be considered in interpreting the findings. A first limitation was the cross-sectional design of the study which hampers definite inference regarding causal relations. A second limitation of our study was that only cognitive social capital was captured and no other common aspects as structural, bonding and bridging social capital. This reduces full comprehensibility of how social capital interacts with sport participation, physical activity and mental health. However, to reduce complexity of the model only cognitive social capital was incorporated in the model as this type has been most consistently related to positive mental health. Furthermore, it should be noted that results of this manuscript only apply to disadvantaged communities and future studies should investigate whether these results can be generalized to other, more prosperous communities. For instance other studies have indicated that communities with low population density are better connected and more civically engaged, compared to communities with high population density. However, these differences were not associated with health outcomes [78].

### Implications

This study answers the call of Eime et al. to investigate how sport, physical, social and psychological outcomes are associated [6]. This study has emphasized the importance of sport participation and individual social capital to improve mental health. It further underscored the importance of community social capital to increase levels of physical activity and individual social capital. On a policy level, results of this study suggest that supporting initiatives aiming at bringing the neighbors together with sport might have beneficial effects on a multitude of outcomes. These local sport initiatives can leverage interest in sport participation which in turn has positive direct effects on physical activity and mental health. Simultaneously these initiatives can excite community social capital that directly affects higher levels of physical activity and individual social capital, leading to better mental health. These results encourage a better interaction among the sport, social and health sector to combine their forces and reach better outcomes in the multidimensional and interrelated concepts of sport participation, physical activity, social capital and mental health. Furthermore, since these results are relevant in a disadvantaged context, a more collaborative approach could be an important strategy to reach better health equity in hard to reach disadvantaged communities.

## Conclusions

This study highlights four important core findings. First, individual social capital is the best predictor of mental health. Second, sport participation and not total physical activity is related with mental health. Third, participating in sport does not improve community or individual social capital in itself; however, engaging in sport with friends, neighbors or families might. Last, community social capital rather than individual social capital predicts higher levels of physical activity. The results of this study imply that cross-sector initiatives between the sport, social and health sector need to be supported as their outcomes are directly linked to one another and can multiply health effects in disadvantaged communities.

## Supporting Information

S1 Appendix Results multiple group analyses. (DOCX)

S2 Appendix Data file in.dat. (DAT)

S3 Appendix Data file in.sav for SPSS. (SAV)

S4 Appendix Input of bootstrapped SEM-analysis executed in M-plus. (INP)

S1 Table Socio-economic characteristics of the selected communities in Antwerp. (DOCX)

S2 Table Socio-demographic characteristics of respondents per community. (DOCX)
