# Supplementary material for: Interrelation of Sport Participation, Physical Activity, Social Capital and Mental Health in Disadvantaged Communities: A SEM-Analysis
Source: PLoS One. 2015 Oct 9;10(10):e0140196. doi: 10.1371/journal.pone.0140196 (PMC4599734; doi:10.1371/journal.pone.0140196)
Supplement: S1 Appendix — (DOCX) [file pone.0140196.s001.docx]

S3 Appendix: Results of multiple group analyses

Multiple group analyses in SEM were executed to verify if relations in our structural model (presented in Fig. 1), differed for male or female respondents, native or ethnic residents, high or low educated people and for other groupings of socio-demographic variables. First, distinct relations were examined for each socio-demographic characteristic. This meant that relationships which were significant for e.g. women but not for man were examined. Second, to assess whether these differences between groups were significant, WALD-tests were completed [[69](#_ENREF_69)]. Results are shown in S4 Table 1 and reported here below.

Results showed two distinct relations between male and female respondents: a) the relation between individual social capital and mental health; b) the relation between community social capital and total physical activity. The relation between individual social capital and mental health was significant for male respondents (β=.246, p<0.001), and not significant for female respondents (β=.112, p>.05). This means that individual social capital had a direct relation with mental health for man and no direct relation was found for women. The relation between community social capital and physical activity was reverse. Female respondents with a higher community social capital showed higher levels of physical activity (β=.160, p<0.05), whereas with male respondents this association could not be detected (β=.018, p>0.05). To know whether these parameters were truly significantly different, the Wald test was performed. This test verifies if the parameters are equivalent between the two groups. Wald test values for the relation of individual social capital and mental health were not significant (value = 1.656; df =1; p >.05), nor were they for the relation of community social capital and physical activity (value = 1.518; df=1; p>.05), meaning that the parameters were the same and that the structural model holds up for both male as female adults.

Results showed three distinct relations between native and ethnic respondents: a) the relation between individual social capital and mental health; b) the relation between community social capital and mental health ; c) the relation between individual social capital and total physical activity. The relation between individual social capital and mental health was significant for native respondents (β=.171, p<0.01), and not significant for ethnic respondents (β=.130, p>.05). This means that individual social capital had a direct relation with mental health for native respondents and no direct relation was found for ethnic respondents. The same relation was found for community social capital and mental health. Native majority members with a higher community social capital showed higher levels of mental health (β=.221, p<0.01), whereas with ethnic minorities this association could not be detected (β=-.029, p>0.05). Last, the relation between individual social capital and total physical activity was significant for native respondents (β=-.152 p<0.05), and not significant for ethnic respondents (β=.023, p>.05). Remarkably, higher levels of individual social capital were associated with lower levels of total physical activity for native respondents, while no such relation was found for ethnic respondents. To know whether these parameters were significantly different, the Wald test was performed. Wald test values for the relation of individual social capital and mental health were not significant (value = .388; df =1; p >.05), they were significant for the relation of community social capital with mental health (value = 7.678; df=1; p<.01), but not for the relation of individual social capital and total physical activity (value = 3.320; df=1; p>.05), meaning that the parameters were the same and that the structural model holds up for both native as ethnic adults except for the relation between community social capital and mental health. Results show that higher levels of community social capital lead to better mental health for native residents, whereas for ethnic residents this was not the case.

Results showed two distinct relations between people of high and low education: a) community social capital and mental health; b) individual social capital and mental health. The relation of community social capital with mental health was not significant for people of high education (β=.040, p>.05). Meaning that higher levels of community social capital of high educated people did not results in higher levels of mental health. Mental health of low educated people (β=.138, p<.05) did increase with higher levels of community social capital. The relation of individual social capital with mental health was significant for high educated people (β=.221, p<.01), not for low educated people (β=.091, p>.05). Indicating that only respondents of high education were benefitting mentally from an increased level of individual social capital. To know whether these parameters were truly significantly different, the Wald test was performed. Wald test values for the relation of community social capital and mental health were not significant (value = 0.844; df =1; p >.05), nor were they for the relation of individual social capital and mental health (value = 2.073; df=1; p>.05), meaning that the parameters were the same and that the structural model holds up for both high as low education people.

Results showed two distinct relations between people with stable partner and without: a) the relation between individual social capital and mental health; b) the relation between community social capital and total physical activity. The relation between individual social capital and mental health was significant for respondents with a stable partner (β=.189, p<0.01), but not significant for single respondents (β=.112, p>.05). This means that individual social capital had a direct relation with mental health for people having a stable partner and no such relation was found for singles. The relation between community social capital and total physical activity was the same. Married respondents or those having a partner with a higher community social capital showed higher levels of physical activity (β=.119, p<0.05), whereas with single respondents this association could not be detected (β=.071, p>0.05). To know whether these parameters were truly significantly different, the Wald test was performed. Wald test values for the relation of individual social capital and mental health were not significant (value = .380; df =1; p >.05), nor were they for the relation of community social capital and total physical activity (value = .362; df=1; p>.05), meaning that the parameters were the same and that the structural model holds up for both adults with and without a stable partner.

Results showed three distinct relations between respondents owning a house and renting a house: a) the relation between individual social capital and mental health; b) the relation between community social capital and total physical activity ; c) the relation between individual social capital and total physical activity. The relation between individual social capital and mental health was significant for owners (β=.166, p<0.01), but not significant for renters (β=.138, p>.05). This means that individual social capital had a direct relation with mental health for owners, whereas no direct relation was found for renters. The same relation was found for community social capital and total physical activity. Owners with a higher community social capital showed higher levels of total physical activity (β=.121, p<0.05), whereas with ethnic minorities this association could not be detected (β=-.047, p>0.05). Last, the relation between individual social capital and total physical activity was significant for respondents owning a house (β=--.120 p<0.05), and not significant for respondents renting a house (β=.023, p>.05). Remarkably, higher levels of individual social capital were associated with lower levels of total physical activity for owners, while no such relation was found for renters. To know whether these parameters were significantly different, the Wald test was performed. Wald test values for the relation of individual social capital and mental health were not significant (value = .104; df =1; p >.05), they were also not significant for the relation of community social capital with total physical activity (value = .762; df=1; p>.05), nor for the relation of individual social capital and total physical activity (value = 2.337; df=1; p>.05), meaning that the parameters were the same and that the structural model holds up for both adults owning and renting a house.

Results showed four distinct relations between young and older adults and a)individual social capital and mental health; b) sport participation and mental health; c) individual social capital and total physical activity; d) sport participation and individual social capital. The relation of individual social capital with mental health was not significant for younger people (β=.122, p>.05), it was for older people. Meaning that higher levels of individual social capital of young adults did not results in higher levels of mental health. Mental health of older adults (β=.172, p<.05) did increase with higher levels of individual social capital. The relation of sport participation with mental health was significant for older respondents (β=.177, p<.01), but not for younger respondents (β=.009, p>.05). Indicating that older adults were benefitting mentally from an increased level of sport participation. The reverse relation was found for individual social capital and total physical activity. Younger adults with a lower individual social capital showed higher levels of total physical activity (β=-.146, p<0.05), whereas with older adults this association could not be detected (β=-.045, p>0.05). Last, the relation between sport participation and individual social capital was significant for older respondents (β=.143 p<0.05), and not significant for younger respondents (β=-.117, p>.05). Meaning that only older adults showed higher individual social capital with higher levels of sport participation. To know whether these parameters were truly significantly different, the Wald test was executed. Wald test values for the relation of individual social capital and mental health were not significant (value = 0.317; df =1; p >.05), nor were they for the relation of sport participation and mental health (value = 3.453; df=1; p>.05), they were also not significant for the relation of community social capital with total physical activity (value = .754; df=1; p>.05), however it was for the relation of sport participation and individual social capital (value = 7.755; df=1; p<.01). Meaning that the parameters were the same and that the structural model holds up for young and older adults except for the relation of sport participation and individual social capital. Findings proved that higher levels of sport participation lead to better individual social capital for older residents, whereas for younger residents this is not the case.

**S4 Table 1**. **Multiple group analysis of different socio-demographic variables.**

| **Socio-demographics** |  | **Community Social Capital** | **Individual social capital** | **Physical Activity** | **Mental Health** |
| --- | --- | --- | --- | --- | --- |
| **Gender** |  |  |  |  |  |
| Male | **Sport Participation** | .036 | .034 | .244*** | .065 |
|  | **Community Social Capital** | - | .231** | .018 | .071 |
|  | **Individual social capital** | - | - | -.050 | .246*** |
|  | **Physical Activity** | - | - | - | -.009 |
| Female | **Sport Participation** | .064 | .019 | .214** | .120 |
|  | **Community Social Capital** | - | .289*** | .160* | .112 |
|  | **Individual social capital** | - | - | -.113 | .126 |
|  | **Physical Activity** | - | - | - | .034 |
| **Ethnicity** |  |  |  |  |  |
| Native | **Sport Participation** | .081 | .037 | .204** | .105 |
|  | **Community Social Capital** | - | .293*** | .134 | .221** |
|  | **Individual social capital** | - | - | -.152* | .171** |
|  | **Physical Activity** | - | - | - | .032 |
| Ethnic | **Sport Participation** | .018 | -.021 | .271*** | .068 |
|  | **Community Social Capital** | - | .206** | .071 | -.029 |
|  | **Individual social capital** | - | - | .023 | .130 |
|  | **Physical Activity** | - | - | - | .000 |
| **Education** |  |  |  |  |  |
| High | **Sport Participation** | .054 | -.045 | .155* | .085 |
|  | **Community Social Capital** | - | .174* | .134 | .040 |
|  | **Individual social capital** | - | - | -.086 | .221** |
|  | **Physical Activity** | - | - | - | .034 |
| Low | **Sport Participation** | .039 | .043 | .332** | .095 |
|  | **Community Social Capital** | - | .265*** | .100 | .138* |
|  | **Individual social capital** | - | - | .039 | .091 |
|  |  | - | - | - | .037 |
| **Age** |  |  |  |  |  |
| Young adults (18-37) | **Sport Participation** | .039 | -.117 | .255*** | .009 |
|  | **Community Social Capital** | - | .184** | .048 | .094 |
|  | **Individual social capital** | - | - | -.146* | .122 |
|  | **Physical Activity** | - | - | - | -.056 |
| Older adults (38-56) | **Sport Participation** | .088 | .143* | .184** | .177** |
|  | **Community Social Capital** | - | .333*** | .137 | .113 |
|  | **Individual social capital** | - | - | -.045 | .172* |
|  | **Physical Activity** | - | - | - | .056 |
| **Tenancy** |  |  |  |  |  |
| Owner | **Sport Participation** | .045 | -.058 | .215*** | .099 |
|  | **Community Social Capital** | - | .180** | .121* | .095 |
|  | **Individual social capital** | - | - | -.120* | .166** |
|  | **Physical Activity** | - | - | - | .020 |
| No owner | **Sport Participation** | .066 | .132 | .240** | .095 |
|  | **Community Social Capital** | - | .313*** | .047 | .075 |
|  | **Individual social capital** | - | - | .023 | .138 |
|  | **Physical Activity** | - | - | - | .024 |
| **Civil Status** |  |  |  |  |  |
| Married / stable partner | **Sport Participation** | .096 | -.011 | .168** | .079 |
|  | **Community Social Capital** | - | .265*** | .119* | .084 |
|  | **Individual social capital** | - | - | -.106 | .189** |
|  | **Physical Activity** | - | - | - | .002 |
| Single | **Sport Participation** | -.019 | .095 | .373*** | .129 |
|  | **Community Social Capital** | - | .247** | .071 | -099 |
|  | **Individual social capital** | - | - | -.024 | .112 |
|  | **Physical Activity** | - | - | - | .084 |
