# Supplementary material for: Interrelation of Sport Participation, Physical Activity, Social Capital and Mental Health in Disadvantaged Communities: A SEM-Analysis
Source: PLoS One. 2015 Oct 9;10(10):e0140196. doi: 10.1371/journal.pone.0140196 (PMC4599734; doi:10.1371/journal.pone.0140196)
Supplement: S1 Table — (DOCX) [file pone.0140196.s005.docx]

**S1 Table**. **The socio-economic characteristics of the selected communities and Antwerp (data of Public Service of Antwerp).**

|  | **Ethnicity rate (%)** | [**Unemployment**](javascript:varinfo(159)) **rate (%)** | [**Average**](javascript:varinfo(1962)) **income (€)** | **Population density (**[**in**](javascript:varinfo(1278))**habitants/km²)** |
| --- | --- | --- | --- | --- |
| **Community** |  |  |  |  |
| A | 65.90 | 18.87 | 15,118 | 14,370 |
| B | 64.60 | 15.20 | 16,464 | 19,150 |
| [C](javascript:geoinfo(5,57)) | 55.06 | 11.93 | 17,891 | 16,818 |
| D | 41.42 | 9.55 | 20,842 | 8,329 |
| E | 39.86 | 12.00 | 16,084 | 16,778 |
| F | 68.71 | 10.06 | 14,819 | 13,577 |
| G | 50.46 | 8.59 | 17,036 | 14,216 |
| H | 47.23 | 11.11 | 18,572 | 15,328 |
| I | 42.62 | 12.22 | 20,880 | 10,751 |
|  |  |  |  |  |
| **Antwerp** | **42.10** | **10.70** | **19,310** | **2,919** |
