# Supplementary material for: Interrelation of Sport Participation, Physical Activity, Social Capital and Mental Health in Disadvantaged Communities: A SEM-Analysis
Source: PLoS One. 2015 Oct 9;10(10):e0140196. doi: 10.1371/journal.pone.0140196 (PMC4599734; doi:10.1371/journal.pone.0140196)
Supplement: S2 Table — (DOCX) [file pone.0140196.s006.docx]

**S2 Table.** **Socio-demographic characteristics of respondents per community.**

| **Groups** | **n (%)** | **Community A** | **Community B** | **Community C** | **Community D** | **Community E** | **Community F** | **Community G** | **Community H** | **Community I** | **Chi square** |
| --- | --- | --- | --- | --- | --- | --- | --- | --- | --- | --- | --- |
| **n** |  | 46 | 45 | 46 | 47 | 46 | 45 | 48 | 46 | 45 |  |
| **Age group** |  |  |  |  |  |  |  |  |  |  |  |
| Young adults (18-37) | 201 (48.6) | 28 (60.9) | 21(46.7) | 20 (43.5) | 18 (38.3) | 18 (40.0) | 21 (45.7) | 27 (56.3) | 27 (58.7) | 21 (46.7) | 0.274 |
| Older adults (38- 56) | 213 (51.4) | 18 (39.1) | 24 (53.3) | 26 (56.5) | 29 (61.7) | 27 (60.0) | 25 (54.3) | 21 (43.8) | 19 (41.3) | 24 (53.3) |  |
| **Gender** |  |  |  |  |  |  |  |  |  |  |  |
| Men | 189 (45.6) | 26 (56.5) | 19 (42.2) | 23 (50.0) | 23 (48.9) | 18 (39.1) | 23 (51.1) | 21 43.8) | 18 (39.1) | 18 (40.0) | 0.678 |
| Women | 225 (54.4) | 20 (43.5) | 26 (57.8) | 23 (50.0) | 24 (51.1) | 28 (60.9) | 22 48.9) | 27 (56.3) | 28 (60.9) | 27 (60.0) |  |
| **Ethnicity** |  |  |  |  |  |  |  |  |  |  |  |
| Native (parents born in Belgium) | 222 (53.6) | 24 (52.2) | 12 (26.7) | 28 (60.9) | 30 (63.8) | 22 47.8) | 20 (44.4) | 32 (66.7) | 29 (63.0) | 25 (55.6) | 0.003** |
| Ethnic (parents born   abroad) | 192 (46.3) | 22 (47.8) | 33 (73.3) | 18 (39.1) | 17 (36.2) | 24 (52.2) | 25 (55.6) | 16 (33.3) | 17 (37.0) | 20 (44.4) |  |
| **Education** |  |  |  |  |  |  |  |  |  |  |  |
| College, university | 194 (46.9) | 11 (23.9) | 18 (40.0) | 20 (43.5) | 18 (38.3) | 24 (52.2) | 17 (37.8) | 35 (72.9) | 24 (52.2) | 27 (60.0) | .000*** |
| Primary, secondary | 220 (53.1) | 35 (76.1) | 27 (60.0) | 26 (56.5) | 29 (61.7) | 22 (47.8) | 28 (62.2) | 13 (27.1) | 22 (47.8) | 18 (40.0) |  |
| **Tenancy** |  |  |  |  |  |  |  |  |  |  |  |
| Owner | 267 (64.5) | 27 (58.7) | 25 (55.6) | 37 (80.4) | 43 (91.5) | 17 (373.0) | 24 (53.3) | 38 (79.2) | 35 (76.1) | 21 (46.7) | .000*** |
| No owner | 147 (35.5) | 19 (41.3) | 20 (44.4) | 9 (19.6) | 4 (8.5) | 29 (63.0) | 21 (46.7) | 10 (20.8) | 11 (23.9) | 24 (53.3) |  |
| **Civil Status** |  |  |  |  |  |  |  |  |  |  |  |
| Married / stable   partner | 290 (70.0) | 27 (58.7) | 32 (71.1) | 39 (80.4) | 29 (63.0) | 30 (66.7) | 32 (66.7) | 28 (60.9) | 37 (82.2) | 29 (63.0) | 0.071 |
| Single | 124 (30.0) | 19 (41.3) | 13 (28.9) | 9 (19.6) | 17 (37.0) | 15 (33.3) | 16 (33.3) | 18 (39.1) | 8 (17.8) | 17 (37.0) |  |

^+^p<0.10, *p<0.05, **p<0.01; ***p<0.001
